# Bee Venom, Honey, and Royal Jelly in the Treatment of Bacterial Infections of the Oral Cavity: A Review

**DOI:** 10.3390/life11121311

**Published:** 2021-11-28

**Authors:** Michał Otręba, Łukasz Marek, Natalia Tyczyńska, Jerzy Stojko, Anna Rzepecka-Stojko

**Affiliations:** 1 Department of Drug Technology, Faculty of Pharmaceutical Sciences in Sosnowiec, Medical University of Silesia in Katowice, Jednosci 8, 41-200 Sosnowiec, Poland; nataliatyczynska1@gmail.com (N.T.); annastojko@sum.edu.pl (A.R.-S.); 2 Department of Instrumental Analysis, Faculty of Pharmaceutical Sciences in Sosnowiec, Medical University of Silesia in Katowice, Jednosci 8, 41-200 Sosnowiec, Poland; lmarek@sum.edu.pl; 3 Department of Toxicology and Bioanalysis, Faculty of Pharmaceutical Sciences in Sosnowiec, Medical University of Silesia in Katowice, Ostrogorska 30, 41-200 Sosnowiec, Poland; jstojko@sum.edu.pl

**Keywords:** honey, royal jelly, bee venom, anti-microbial activity, oral cavity bacteria

## Abstract

Oral diseases affect a very large number of people, and the applied pharmacological methods of treatment and/or prevention have serious side effects. Therefore, it is necessary to search for new, safer methods of treatment. Natural bee products, such as honey, royal jelly, and bee venom, can be a promising alternative in the treatment of oral cavity bacterial infections. Thus, we performed an extensive literature search to find and summarize all articles about the antibacterial activity of honey, royal jelly, and bee venom. Our analysis showed that these bee products have strong activity against the bacterial strains causing caries, periodontitis, gingivitis, pharyngitis, recurrent aphthous ulcers, supragingival, and subgingival plaque. An analysis of average MIC values showed that honey and royal jelly have the highest antimicrobial activity against *Porphyromonas gingivalis* and *Fusobacterium nucleatum*. In turn, bee venom has an antibacterial effect against *Streptococcus mutans*. *Streptococcus sobrinus* and *Streptoccus pyogenes* were the most resistant species to different types of honey, and royal jelly, respectively. Moreover, these products are safer in comparison to the chemical compounds used in the treatment of oral cavity bacterial infections. Since the antimicrobial activity of bee products depends on their chemical composition, more research is needed to standardize the composition of these compounds before they could be used in the treatment of oral cavity bacterial infections.

## 1. Introduction

According to the article published by WHO in March 2020, oral diseases affect nearly 3.5 billion people all over the world. Moreover, more than 530 million children suffer from dental caries of deciduous teeth [1]. It is noteworthy that in 2016 periodontal disease was the 11th most prevalent condition in the world. This is a very important fact since periodontal disease is the major cause of tooth loss and can affect the whole body health. Furthermore, the spreading of microorganisms and their products from periodontal tissues may cause inflammation in other organs of the body. Thus, treating oral diseases is crucial [2].

The oral microflora consists of over 700 species of gram-negative and gram-positive bacteria, both aerobic and anaerobic ones [3]. Microorganisms living in the oral cavity on the tooth surface have the form of two types of biofilm: supra- and subgingival one, differing significantly in the composition of the bacterial flora. The supragingival biofilm causes caries, while the subgingival one causes gingivitis and periodontal disease [4]. The main bacterial strains causing the oral cavity microbial infections are presented in Table 1.

As presented in Table 1, there are many ways of treating the oral diseases caused by bacterial infections, such as periodontitis, gingivitis, pharyngitis, and recurrent aphthous ulcers (RAS). Unfortunately, the treatment of the microbial infections of the oral cavity as well as the prevention of caries can cause many side effects listed in Table 1. Bee products, such as bee venom, honey, and royal jelly, also cause side effects (allergies, which may additionally lead to local inflammation and hemolysis), but they appear only when high concentrations of a given product are used [22]. Moreover, the single component of bee venom, like mellitin, possesses a strong toxic effect, while total bee venom causes only the presence of local contact allergies. It is noteworthy that the effect is not observed during the application of bee venom [23]. Interestingly, the side effects observed after pharmacological treatment and/or prevention (discoloration of the teeth and tongue, exfoliative stomatitis, swelling of the parotid salivary glands, enlargement of the filamentous papillae of the tongue, and taste disturbances) are not observed after the use of bee products [24,25].

The etiology factors of dental carries include bacteria, time, susceptible tooth surface, and fermentable carbohydrates. The risk of dental caries may also increase due to sociodemographic factors, such as poor oral hygiene, age, improper tooth brushing habits, plaque, and sugar-containing drinks [26]. Eating carbohydrates and sugars increases acid production by bacteria and other organisms, which leads to cavities, gingivitis, and other forms of tooth decay caused by plaque [27]. For gingivitis, the risk factors are as follows: not maintaining oral hygiene, smoking or chewing tobacco, genetic factors, misaligned teeth that are difficult to clean, stress, lack of nutrients, puberty, pregnancy, hormone changes, diabetes, HIV, and some medications (steroids or cancer therapy drugs) [27]. Pharyngitis may be caused by viral and bacterial agents, such as measles, adenovirus, chickenpox, croup (childhood illness with a characteristic barking cough), whooping cough, and group A of *Streptococcus* [27]. Periodontitis may also be caused by poor oral hygiene as well as smoking, type 2 diabetes, obesity, hormonal changes in women (during menstruation, pregnancy, or menopause) that can make the gums more sensitive, HIV or leukemia, medications that reduce the flow of saliva in the mouth, genetics, and poor nutrition (deficiency in vitamin C) [27]. Recurrent aphthous ulcers (RAS) may be caused by trauma to the oral mucosa due to local anesthetic injections, sharp teeth, dental treatments, and toothbrush injury [19].

Bee venom is secreted by venom glands of the honeybee (*Apis mellifera*) and contains the venom and pheromones. The dry matter of the venom includes peptides (melittin, apamin, peptide 401-degranulating mast cells, secapin, adolapin, and procamine) and proteins with enzymatic properties (phospholipase A2, hyaluronidase, phosphodiesterase, and lysophospholipase). In addition, there are low molecular weight compounds in the venom (pheromones, biogenic amines, sugars, amino acids, phospholipids, and bioelements). The rich composition of the venom provides multidirectional effects, including analgesic and anti-inflammatory effects [28,29]. Bee venom also contains antibacterial proteins, namely phospholipase A2, hyaluronidase, and melectin [30].

Honey is produced by bees from nectar in a series of complex chemical and physical reactions that increase the sugar content. The composition of honey depends on where and when it is harvested, how the ecosystem functions, and how honey is stored. Consequently, there may be differences in the antibacterial properties of honey [31]. Honey is rich in biologically active compounds: carbohydrates, vitamins (B, C, E, and carotenoids), enzymes (invertase, glucose oxidase, and lysozyme), amino acids, fatty acids, essential oils, and hormones. Moreover, honey contains many valuable macro- and microelements, such as Ca, Fe, Co, Mg, Mn, F, Zn, Na, and K. Additionally, honey contains peptides: defensin-1 and the main royal jelly protein 1 (MRJP1), and both of these compounds exhibit strong antibacterial activity [28,32].

Royal jelly is secreted by pharyngeal glands in front of the head of worker bees. Initially, it has the form of a white, liquid substance with creamy consistency. The milk tastes tart and sour, its pH is low from 3.4 to 4.3, and its aroma is sharp and characteristic [33]. Proteins constitute about 17–45% of the dry matter of milk, and the milk is also rich in enzymes (proteinases, acid phosphatase, glucose oxidase, α-amylase, catalase, α-glucosidases, and glucocerebrosidases) and hormones (neurohormone-acetylcholine, progesterone, and testosterone). Major royal jelly proteins (MRJP) are also an important component of the milk because they have a strong antibacterial effect [28,34].

The aim of writing this manuscript is to summarize up-to-date research on bee products influence on the treatment of oral cavity bacterial infections. Since the antibacterial activity of propolis against different bacterial strains is well and frequently described in other reviews [35,36,37,38], in this review, we focused on bee venom, honey, and royal jelly as possible candidates for use in maintaining oral hygiene and therapy of bacterial infections of the oral cavity. Thus, we mainly focused on the in-vitro assays using the bacterial strains responsible for oral cavity diseases and related to maintaining oral hygiene, and only few in-vivo studies were analyzed.

## 2. Materials and Methods

**Figure 1:**
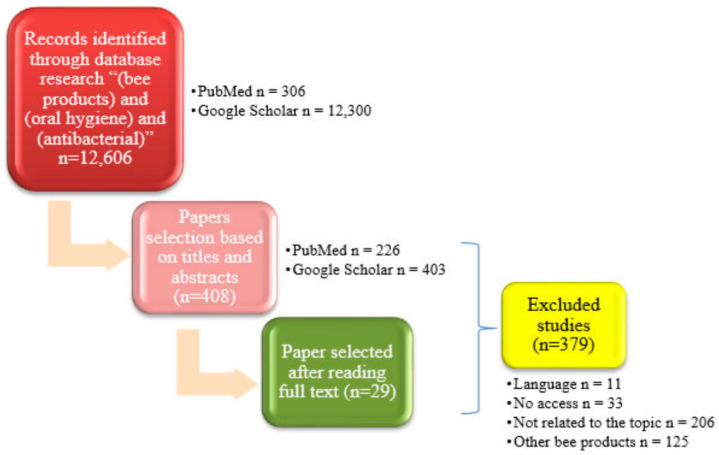
The selection and result of the papers.

PubMed and Google Scholar were used in September 2020 to search for English-language papers containing phrases “(bee products) and (oral hygiene) and (antibacterial).” 12,300 records from Google Scholar and 306 records from PubMed were found, from which 407 records were classified for further analysis based on paper titles and abstracts published between 1983 and 2021. Then, 379 papers were rejected. The exclusion selection criteria were (1) no access, (2) another language than English, (3) review papers, (4) lack of information about the antibacterial activity of bee products in the manuscript, and (5) main focus on propolis in the manuscript or bee products that do not possess antibacterial activity against the bacterial strains causing oral cavity infections. The criteria of inclusion selection were (1) peer-reviewed journals in English containing information about the in-vitro studies of bacterial strains causing oral cavity infections and (2) in-vivo studies showing the use of bee products in maintaining oral hygiene. At the final stage, we decided to add one publication from 2021. The remaining articles were divided into 4 groups based on the type of the tested bee product: there were 23 papers about honey, 2 about bee venom, 2 about royal jelly, and 2 about the mixtures (Figure 1). The papers that were selected include 29 original articles published between 1994 and 2021.

The methodological limitations to be considered: it is possible that other databases would provide more articles, but even if they did, the differences in the number of papers would be minimal because PubMed is the gold standard of searching, and Google Scholar uses one of the largest indexing engines.

## 3. Antibacterial Activity of Bee Products in Oral Cavity Bacterial Infections

### 3.1. Honey

Honey is useful in oro-dental care, especially in the treatment of dental plaque, gingivitis, and mitigating malodor [39]. Moreover, scientific studies have shown the antibacterial activity of honey against gram-positive and gram-negative bacteria, including *S. mutans* [40,41]. It is noteworthy that the mechanism of action is different from the one of antibiotics—honey stops bacterial growth due to high sugar content and low pH (bacteriostatic action) and kills bacteria with hydrogen peroxide and other antibacterial agents. In the case of dental plaque, Manuka honey effectively prevents biofilm growth as well as reduces the amount of produced acids [42]. Moreover, honey has an anti-plaque, anti-calculus effect, and it may be used post-operationally in the extraction socket and the treatment of mouth ulcers, recurrent herpes labialis, or lichen planus due to its various activities [43]. Honey is also useful in periodontal disease, inflammation of mucous membranes in the mouth (stomatitis), and the treatment of halitosis, as well as mouth ulcers, and for periodontitis prevention [10].

The antimicrobial activity of honey from the blossoms of Eucalyptus trees against *S. oralis*, *S. anginosus*, *S.* *gordonii*, *S. salivarius*, *S. mutans*, *S. sanguinis,* and *S. sobrinus* was evaluated by Basson et al. (1994). The MIC values against all tested strains ranged from 12% to 25% (Table 2). The results indicated that the tested honey did not exhibit high antimicrobial activity [39].asson et al. (2008) analyzed the antimicrobial activity of various types of honey produced from plants growing in South Africa (honey produced from the blossoms of *L. cordifolium*, Pincushion honey, and Fynbos honey), Australia (Bluegum honey), and New Zealand (Manuka bush honey) against *S. mutans, S. salivarius, S. sanguinis, S. anginosus, S. gordonii, S. oralis,* and *S. sobrinus*. The obtained MIC values were between 12.5 and 50% (Table 2). The results indicated that the South African samples of honey did not exhibit any exceptionally high antimicrobial activity, but the carbohydrate concentration above 25% played a key role in the antimicrobial activity of the tested types of honey. It was shown also that *S. anginosus* and *S. oralis* were more sensitive than the other microbial species. The authors showed that the types of honey from South Africa did not differ from the types of honey from Australia and New Zealand [5].

The antimicrobial activity of Manuka honey against *Campylobacter* spp. was evaluated by Lin et al. (2009). The authors analyzed the clinical isolates identified with multiplex PCR and type culture collection strains of these species to determine the MIC of Manuka honey. The MIC values against all tested *Campylobacter* (20 strains of *C. jejuni* and seven strains of *C. coli*) ranged from 0.8% to 1.1% (*v*/*v*) of Manuka honey (Table 2) and were similar to the minimum bactericidal concentration (MBC). This indicated that these isolates were sensitive to Manuka honey, which suggests that honey might be useful for treating periodontitis [40].

Tan et al. (2009) compared the antibacterial activity of Tualang honey (TH) with that of Manuka honey against *S. pyogenes*. Manuka honey was used as the control. The obtained MIC and MBC values of Tualang honey were similar to the ones of Manuka honey (Table 2). The results showed that Tualang and Manuka honeys caused similar inhibition of *S. pyogenes* growth [41].

The antimicrobial activity of 60 types of honey of various botanical origins was examined by Voidarou et al. (2011). The authors showed that coniferous, citrus, and thyme types of honey, except for polyfloral honey, which was obtained in bulk from apiarist’s open markets in Epirus province in Greece, exhibited antibacterial activity against *S. pyogenes*. The analysis of the minimum active dilution, defined as the dilution (% *w*/*v*) of honey that causes an inhibition zone of 1 mm in diameter, showed that coniferous and thyme types of honey exhibited the activity against *S. pyogenes* with dilution of 22.38% and 21.78% (*w*/*v*) followed by citrus honey of 27.74% dilution (*w*/*v*) (Table 2) [44].

Badet et al. (2011) investigated the effect of two types of Manuka honey on *S. mutans*, *Actinomyces viscosus,* and *Lactobacillus rhamnosus*. The results showed that Manuka 1 was more efficient than Manuka 2 (data shown in Table 2). The authors also investigated the activity of Manuka types of honey on multi-species biofilm formation (*S. mutans*, *S. sobrinus*, *L. rhamnosus*, *A. viscosus*, *P. gingivalis,* and *F. nucleatum*). Manuka 1 inhibited biofilm formation at the concentration of 200 μg/mL and Manuka 2 at the concentration of 500 μg/mL, which suggests the ability of Manuka honey to protect against biofilm formation [45].

The effect of natural honey on *S. mutans* growth, viability, and biofilm formation was investigated by Nassar et al. (2012). Natural honey was obtained from a local grocery store in Jeddah (Saudi Arabia) and was compared with artificial honey, which contained the same amount of sugars. The suggested MIC value for natural honey was between 25 and 12.5% (Table 2). At 12.5%, natural honey was less encouraging to bacterial growth and biofilm formation than artificial honey, which suggests that the content of sugar is not the only factor responsible for the antimicrobial activity of honey [46].

The antibacterial efficacy of honey against oral bacteria (*S. mutans*, *C. rectus, S. sanguinis, A. actinomycetemcomitans,* and *P. gingivalis*) was evaluated by Aparna et al. (2012). Moreover, the authors compared the antiplaque efficacy of honey with the one of chlorhexidine in vivo. In the in-vitro part, the inhibitory effects of 0.2% chlorhexidine gluconate, honey mouthwash, and saline against the oral bacteria were analyzed. The authors also obtained MIC values for honey mouthwash (Table 2) and for 0.2% chlorhexidine gluconate (1:2, 4, 1:16, 1:2, 1:16 μg/mL, respectively) for *S. mutans, A. actinomycetemcomitans, P. gingivalis, C. rectus*, and *S. sanguinis*. It was shown that honey mouthrinse effectively inhibited the tested strains. However, the chlorhexidine gluconate rinse had the lowest MICs, and saline had no inhibitory effect. In the in-vivo part, participants were divided into three groups of 22 each (group 1 = chlorhexidine; group 2 = honey; group 3 = saline), and the plaque scores were compared at baseline after four days. The in-vivo results revealed that plaque formation was reduced by chlorhexidine and honey rinses. It suggests that the honey is effective against oral bacteria and reduces plaque formation [47].

Ahmadi-Motamayel et al. (2013) evaluated the antibacterial activity of honey obtained directly from Hamadan (Iran) beekeepers on *S. mutans* and *Lactobacillus* spp. The results indicated that natural honey exhibited an antibacterial activity on *S. mutans* in concentration higher than 20%, and with 100% concentration on *Lactobacillus* spp. [48].

Eick et al. (2014) analyzed the effect of a local German multifloral blossoms honey from a beekeeper and a New Zealand Manuka honey and the effect of hydrogen peroxide and methylglyoxal (both 5, 20, and 100 mg/L) on *P. gingivalis*. Results indicated that Manuka honey (2%) and the German beekeeper honey (5%) inhibited the growth of 50% of *P. gingivalis* (MIC_50_), as shown in Table 2. The MIC values were 5–20 mg/L for methylglyoxal and 10–100 mg/L for hydrogen peroxide. The authors indicated that the Manuka honey contained 1.87 mg/kg of hydrogen peroxide and 982 mg/kg of methylglyoxal. On the other hand, in German honey, there are 3.74 mg/kg and 2 mg/kg, respectively. The authors concluded that honey exhibited antibacterial activity against *P. gingivalis* and that the same effects of Manuka honey could be attributed to methylglyoxal. It was also shown that 10% of both types of honey inhibited but not prevented the formation of *P. gingivalis* biofilms and reduced the numbers of viable bacteria in 42-h-old biofilms, so they may have potential in prevention and treatment of periodontitis [49].

The effects of chewing honey on plaque formation and dental plaque bacterial counts were investigated by Atwa et al. (2014). The authors also determined whether honey possessed antibacterial effects against *S. mutans*, *L. acidophilus*, and *P. gingivalis* recovered from plaques, which were collected from 20 female orthodontic patients. After chewing honey or rinsing the oral cavity with 10% sucrose or 10% sorbitol, the pH of plaque was measured, and the numbers of tested strains in respective plaques were determined. It was shown that bacterial counts were significantly reduced in the honey group compared to sucrose and sorbitol groups (positive and negative controls, respectively). Moreover, the honey significantly inhibited the growth of all studied strains when compared to inhibition observed with the use of antibiotics (*p* < 0.001) (27.6 mm for *S. mutans*, 24.5 mm for *P. gingivalis,* 31.9 mm for *L. acidophilus*). The authors concluded that topical application of honey could modify the pH, reduce bacterial counts, and inhibit bacterial growth, and therefore, honey might be useful in the prevention of gingivitis and caries in patients undergoing orthodontic treatment [50].

Schmidlin et al. (2014) evaluated the antibacterial efficacy of different Manuka honey products against *S. mutans*, *P. gingivalis,* and *A. actinomycetemcomitans*. The authors tested the types of Manuka honey with different non-peroxide activity (NPA) values. The MIC values of Manuka honey 25+ against *A. actinomycetemcomitans*, *P. gingivalis*, and *S. mutans* are shown in Table 2. The lowest dilution required to kill these bacteria was >1:2 for all three bacterial strains. The results showed that Manuka honey had an NPA value- and dose-dependent antibacterial efficacy [51].

Nishio et al. (2016) tested a combination of two types of stingless bee honey produced by *Scaptotrigona bipunctata* (SB) and *Scaptotrigona postica* (SP) against *Streptococcus* spp. Inhibition zones generated by SB and SP honey samples were 19 and 11 mm against *S. mutans* as well as 14 and 8 mm against *S. pyogenes*. The MIC values are shown in Table 2. The results revealed that natural honey possessed in-vitro antimicrobial activity against *S. mutans* and *S. pyogenes* [52].

The antimicrobial activity of honey from Morocco against *S. pyogenes* was evaluated by Benlyas et al. (2016). The authors analyzed 11 different types of honey: acacia, carob, eucalyptus, harmal, jujube, lavender, orange, reseda, rosemary, spurge, and thyme honeys. The MIC values against *S. pyogenes* ranged from 7.33 to 11 mg/mL (Table 2). Interestingly, the analysis of the disc inhibition zone showed that *S. pyogenes* was resistant to gentamycin (10μg/disc). The values of disc inhibition zone were 21.69 ± 0.58, 16,71 ± 0.89, 15.53 ± 0.46, 17.62 ± 0.52, 18.93 ± 0.52, 14.80 ± 0.85, 12.21 ± 0.31, 13.56 ± 0.62, 18.37 ± 0.96, 20.44 ± 0.54, and 23.52 ± 0.71 mm (*p* < 0.001), respectively, for acacia, carob, eucalyptus, harmal, jujube, lavender, orange, reseda, rosemary, spurge, and thyme honey. The study showed that Moroccan honey exhibited antimicrobial activity, which is in high correlation with their phenolic and flavonoid content. This suggests that honey may be used in the treatment of oral infectious diseases. Moreover, it may be used as an alternative to antimicrobial drugs [53].

Safii et al. (2017) assessed the antibacterial activity of Manuka honey against plaque-associated bacteria in vitro (*S. mutans*, *F. nucleatum*, and *P. gingivalis*). The obtained MIC values ranged from 6.3% to 25% (Table 2), whereas the MBCs were from 12.5% to 50% (*w*/*v*). Results showed that *S. mutans* was the most resistant one. Manuka honey is antimicrobial towards representative oral bacteria. However, the authors concluded that despite its antibacterial activity, Manuka honey could not be used as an adjuvant in the treatment of periodontal disease because it causes demineralization of oral hard tissues at natural pH and due to the relative resistance of *S.mutans* related to the high concentrations of fermentable carbohydrates in the honey [54].

The antibacterial activity of Manuka honey against *S. mutans* and *Lactobacillus* spp. were analyzed by Beena et al. (2018). The authors tried to compare the efficacy of Manuka honey with Dabur honey. The results showed that Manuka honey (100% and 25%) has a statistically significant (*p* ≤ 0.001) antibacterial effect against *S. mutans* and *Lactobacillus* spp. in comparison to the Dabur honey (100% and 25%). Moreover, both types of honey in the concentration of 100% showed a statistically significant (*p* ≤ 0.001) stronger antibacterial effect against *S. mutans* and *Lactobacillus* spp. in comparison to the concentration of 25%. It suggests that Manuka honey exhibited more antibacterial activity against the tested bacteria than Dabur honey [55].

Habluetzel et al. (2018) analyzed the antimicrobial activity of Swiss, German, and Manuka honeys against *S. gordonii*, *S. sanguinis*, *S. mutans*, *S. sobrinus*, *L. acidophilus*, and *A. naeslundii*. Moreover, they analyzed the two components of honey such as methylglyoxal (40% water solution) and hydrogen peroxide (2 mM). The erosion experiment showed that Manuka honey significantly (*p* = 0.023) increased surface hardness at 30, 4, and 30 min of pellicle modification, while Swiss midland honey significantly (*p* = 0.008, and *p* = 0.003) increased surface hardness at 4 and 30 min. In contrast, methylglyoxal decreased surface hardness even without erosion challenge at 30 min (*p* = 0.011). Moreover, the adhesion study showed that 2 h and 30 min incubation with Manuka honey significantly (*p* = 0.021) reduced the adherence of *S. gordonii*. Methylglyoxal also significantly (*p* = 0.038) decreased the adherence of *S. gordonii* but only after 2 h. The obtained MIC values showed that the Manuka honey was the most effective against the strains since it inhibited bacterial growth in lower concentrations than Swiss or German honeys (data shown in Table 2). Moreover, Manuka honey inhibited the adhesion of *S. gordonii* to the pellicle, which suggests the protection against bacterial adhesion at an early stage of biofilm formation. The erosion experiment demonstrates that the honey did not cause enamel erosion contrary to the methylglyoxal, which stimulated the process [56].

The antibacterial activity of Spanish honey against *S. pyogenes* was analyzed by Combarros-Fuertes et al. (2019). The authors used 15 different samples of Spanish honey and Manuka honey as a control. The MIC value was similar as in the control for most samples (0.20 g/mL) except for multifloral honey H4a (0.25 g/mL), which was less effective against the *S. pyogenes*. It is noteworthy that multifloral honey H8a was more effective against the strain since the MIC was 0.10 g/mL (Table 2). The analysis of minimal lethal concentration (MLC) showed that most samples had the MLC values similar to the control (0.20 g/mL), while rosemary honey H4, multifloral honey H4a, and thyme honey H7a were less effective against *S. pyogenes* (MLC = 0.25 g/mL). Since the MIC and MLC values were similar, it can be suggested that the analyzed types of honey have not only bacteriostatic but also bactericidal effects. Moreover, the analyzed Spanish honey, except for multifloral H4, may be used in the treatment of oral diseases [57].

The effect of Tualang honey (TH) against *S. pyogenes* was investigated by Al-Kafaween et al. (2020). In this study Tualang honey, which is a Malaysian multifloral jungle honey, was used. The MIC and MBC for TH against *S. pyogenes* were 13% (*w*/*v*) (Table 2) and 25% (*w*/*v*), respectively. It suggests that Tualang honey could be used as an alternative therapeutic agent for microbial infection [58].

Brown et al. (2020) assessed the antimicrobial properties of five honey samples against *S. pyogenes* obtained from three species of bees: two stingless bees (*Frieseomelitta nigra* and *Melipona favosa)* and one stinging bee (*Apis mellifera*). The obtained MIC values are shown in Table 2. However, the obtained MBC values were 4% (*w*/*v*) for *F. nigra* honey from Tobago, 8% for *F. nigra* honey from Trinidad, 16% for *M. favosa* honey from Tobago, and 32% for *A. mellifera* honey from Tobago. The results indicated that stingless bee types of honey from Tobago exhibited the greatest antimicrobial activity [59].

The antibacterial effect of citrus honey, *Saturja* spp. honey, and oregano and sage honey against *S. mutans* and *F. nucleatum* was evaluated by Voidarou et al. (2021). All samples were obtained directly from producers in Greece and compared with Manuka honey and artificial honey. The inhibition zones formed with honey of *Satureja* spp. were 20.7 ± 3.7 mm, 20.1 ± 4.9 mm for oregano and sage, and 18.3 ± 4.7 mm for citrus honey against *S. mutans*, whereas 16.9 ± 2.2, 15.1 ± 2.1, and 11.3 ± 1.3, respectively, against *F. nucleatum*. The obtained MIC values are shown in Table 2. The results indicated that the *Saturja* spp. and the oregano and sage honeys exhibited a greater antibacterial activity against the tested bacteria than Manuka honey [60].

To underline the significant effect of honey in oral cavity bacterial infection treatment, we decided to include some in-vivo studies that we found. The first in-vivo study analyzing the impact of honey on the clinical level of the dental plaque was performed by Jain et al. (2015). Ninety patients were divided into three groups: the honey loaded into the gingival sulcus of all the teeth, the chlorhexidine gluconate (0.2%) mouthwash, and the combination of xylitol chewing gum and chlorhexidine (0.2%) mouthwash used for 15 and 30 days. A decrease in the plaque index was observed in all groups. Moreover, honey significantly (*p* < 0.05) reduced the plaque index in comparison to chlorhexidine and chlorhexidine with xylitol. This suggests that honey may be useful in maintaining oral cavity hygiene [61]. The second study was performed by Singh et al. (2016), who compared an impact of 10% honey and 0.12% chlorhexidine mouthwash on dental plaque levels and gingival health. Thirty participants were divided into two groups of 15 people: the test group using honey and the control group using chlorhexidine for 15 days. The study showed a significantly higher mean plaque index score and the mean papilla bleeding index score of the test group in comparison to the control group (PI ≤ 0.5). The result suggests that chlorhexidine prevents the regrowth of plaque and controls gingival bleeding. Moreover, the analysis of the mean plaque index score showed that chlorhexidine rinse more effectively prevents plaque accumulation than 10% honey [62].

### 3.2. Royal Jelly

Antimicrobial activity of royal jelly towards *A. actinomycetemcomitans*, *P. gingivalis*, *Prevotella intermedia,* and *F. nucleatum* was analyzed by Coutinho et al. (2018). The obtained MIC values were between 0.2 μg/mL and 12.5 μg/mL (Table 3). It is worth noting that *P. gingivalis* and *P. intermedia* were sensitive to royal jelly in the concentration range from 0.2 to 100 μg/mL, while *A. actinomycetemcomitans* and *F. nucleatum* were resistant to royal jelly in the concentration range from 0.2 to 6.25 μg/mL. The obtained MBC values were identical, which may suggest that royal jelly possesses antibacterial activity against periodontal bacteria [63].

The antibacterial activity of the royal jelly against periodontopathic bacteria was evaluated by Khosla et al. (2020). The authors analyzed subgingival plaque samples from the patients. The authors noticed a significant difference (*p* < 0.05) between the colony-forming unit (CFU) of aerobic (55.8 × 10^−4^ CFU) and anaerobic (262.8 × 10^−4^ CFU) bacteria. The aerobic bacteria responsible for periodontitis are *P. gingivalis*, *P. intermedia*, *T. forsythia*, *T. denticola*, and *F. nucleatum*, while *A. actinomycetemcomitans* is a relative anaerobe. Moreover, the MIC of the royal jelly was higher than the one of chlorhexidine (6.25 μg/mL and 3.25 μg/mL, respectively, for aerobic and anaerobic bacteria), which is a gold standard antiplaque agent (data shown in Table 3). It is noteworthy that royal jelly possesses stronger activity against anaerobic bacteria than aerobic ones. The minimum bactericidal concentration showed no growth for chlorhexidine and minimum growth for royal jelly. Moreover, royal jelly is a natural product that has minimal side effects and can work synergistically with other antiplaque agents. Thus, it may be used as an alternative to synthetic antimicrobials [64].

### 3.3. Bee Venom

The antibacterial effect of bee venom against *S. mutans* was analyzed by Kim et al. (2006). The obtained MIC and MBC values were the same Table 4. Moreover, the authors confirmed that *S. mutans* was resistant to 5 of 17 analyzed antimicrobial agents: cephalothin, clindamycin, cefazolin, lincomycin, and penicillin [65].

Antimicrobial activity of apitoxin, melittin, and phospholipase A2 of *Apis mellifera* bee venom against *S. salivarius*, *S. sobrinus*, *S. mutans*, *Streptococcus mitis*, *S. sanguinis*, and *Lactobacillus casei* were analyzed by Leonardo et al. (2015). The obtained MIC values of chlorhexidine were 0.9 μg/mL (for *S*. *salivarius*, *S. sobrinus*, *S. mutans*, and *L. casei*) and 3.7 μg/mL (for *S. sanguinis* and *S. mitis*). The results showed that both apitoxins exhibited similar and good antimicrobial activity. Moreover, melittin was the most potent bee venom compound against all tested strains except *S. mutans*. All tested compounds were less effective than chlorhexidine since the obtained MIC values for the tested compounds were higher than for chlorhexidine (Table 4) [66].

### 3.4. Mixtures

The antibacterial effect of royal jelly as well as toothpaste containing fennel and Yahashi honeys against *S. mutans* and *P. gingivalis* were analyzed by Suzuki and Yamaguchi (2016). The obtained MIC values against *S. mutans* were 25, more than 100, and 100 mg/mL, respectively, for royal jelly, fennel honey, and Yahashi honey. In the case of royal jelly and honey mixture, MIC values were 12.5, 50, and 6.25 mg/mL for royal jelly, fennel honey, and Yahashi honey, respectively. Similar MIC values were obtained by the authors against *P. gingivalis*: 25 and 100 mg/mL, respectively, for royal jelly and Yahashi honey. In the case of royal jelly and Yahashi honey mixture, MIC values were 12.5 and 25 mg/mL, respectively. Therefore, toothpaste containing royal jelly and honey may prevent periodontal diseases [67].

The efficacy of bee products mixture (96.7% honey, 3% royal jelly, and 0.03% propolis) in the infections of the upper respiratory tract (including sore throat) of children from 5 to 12 years of age were analyzed by Seçilmiş and Silici (2020). The mixture was consumed for 10 days (20 g/day for children under 30 kg, 40 g/day for children over 30 kg). The study showed that in the group that received the mixture and antibiotic, bacterial infection significantly (*p* < 0.05) decreased on the second and the fourth day in comparison to the group treated with antibiotics alone. It can be concluded that bee products enhance antimicrobial activity [68].

## 4. Antibacterial Properties of Bee Products: Mode of Action

### 4.1. Honey

Honey has a broad spectrum of antibacterial activity. It can prevent or kill bacteria due to different mechanisms of action, such as high sugar concentration, low pH, hydrogen peroxide generation, proteinaceous compounds, phenolic compounds, or other antimicrobial compounds present in honey [32]. The amount of unbound water is low (water activity values between 0.56 and 0.62) but sufficient to let bacteria grow [32,69]. The high sugar concentration (about 80% *w*/*v*) causes osmotic stress. Hyperosmolar environment leads to bacteria dehydration (water flows out of the bacterial cells) and makes them unable to grow and proliferate [32,70,71]. Moreover, sugars also interfere in bacterial quorum sensing, while osmotic pressure may affect bacteria biofilm formation [70].

Honey is an acidic food since pH is between 3.2 and 4.5, which forms a non-convenient environment for microbial growth [70,71]. Acidity is caused by the organic acids (0.5% *w*/*v*) present in honey, while pH neutral for bacterial growth is from 6.5 to 7.5. However, pH value alone is not enough to inhibit the growth of bacteria diluted in food or bodily liquids [69]. On the other hand, the presence of other antibacterial compounds in honey depends on the botanical origin of the sample [70].

Additionally, enzymes (glucose oxidase and lysozyme) present in honey have antimicrobial effect. Glucose oxidase may inhibit the growth of bacteria; when the enzyme is diluted to 50%, the quantity of gluconic acid and hydrogen peroxide increases [32]. In undiluted honey, the enzyme is not active due to low pH [69]. The maximum H_2_O_2_ concentration is usually reached in the concentration range from 15 [70] or 30 [65] to 50% [69,70]. The hydrogen amount in honey is between 0.5 and 2.5 mM [72]. It is worth noting that light types of honey produce less hydrogen peroxide than the dark ones. The MIC value for hydrogen peroxide is in the range of 10–1000 µg/mL [70]. Moreover, Cu^+^ or Fe^2+^ ions present in honey degrade H_2_O_2_ and produce hydroxyl radicals, which are more responsible for DNA damage than hydrogen peroxide and peroxide [70,72]. On the other hand, hydrolysis of hydrogen peroxide to oxygen leads to polyphenols auto-oxidation, which occurs as pro-oxidant molecules exhibit bacteriostatic and DNA-damaging activities. Furthermore, benzoic acids in different types of honey react with hydrogen peroxide resulting in peroxy-acids, which are stronger antimicrobial compounds than hydrogen peroxide, and they may also resist catalase activity [70]. Interestingly, lysozyme “hydrolyzes the *β*-1,4 linkage between the residues of N-acetylmuramic acid and N-acetyl-D-glucosamine in the peptidoglycan of the bacterial wall” [32].

The antibacterial effect of polyphenols (phenolic acids and flavonoids) present in honeys depends on synergistic effects with other polyphenols or other compounds (H_2_O_2_) since the individual concentration of polyphenols is not sufficient [70].

Methylglyoxal (MGO) is a 1,2-dicarbonyls-breakdown-product, which exhibits antimicrobial activity and is present in Manuka honey. MGO can be found not only in New Zealand and Australia honey but also in the mire and polyfloral Nordic honey that is different monofloral honey varieties (citrus, eucalyptus, acacia, chestnut, lime, rhododendron, strawberry tree, sulla, sunflower, and thyme), honeydew and polyfloral honey from Italy, Finnish polyfloral honey samples, and honeydew Portuguese honey. The concentration of MGO in Manuka honey is in the range from 38 to 1541 mg/kg, while in other types of honey, the concentration range is from 0.2 to 166 mg/kg. The antibacterial activity of methylglyoxal includes alterations in the bacterial fimbriae structure and flagella, which results in a decrease of adherence and motility of bacteria. Moreover, MGO led to damage of cell membranes and the shrinking and rounding of cells [70]. In contrast, many bacteria can detoxify MGO; thus, other components must modulate antibacterial activity [71]. Interestingly, heating of honey to 37 °C results in increased MGO, while overheating it to 50 °C leads to the loss of MGO and dihydroxyacetone [72].

It is worth noting that bee defensin-1 known as royalisin is present in bee hemolymph, royal jelly, as well as in the head and thoracic section of bees [32]. Defensin-1 is in the medical-grade honey Revamil, Manuka honey, honey from several regions of Slovakia, and eucalyptus honey samples from Ecuador [70]. The amount of bee defensin-1 may vary in honey samples [72]. The antibacterial activity involving the pore formation in the bacterial cell membrane leads to cell death by the efflux of important ions and nutrients. Moreover, defensin-1 plays an important role in the antibiofilm activity of honey [70].

The impact of honey on bacterial structural and morphological changes, membrane potential, cell cycle, and cell growth, metabolism, efflux pump activity, cell-cell communication, biofilm inhibition, and stress response were described by Combarros-Fuertes et al. (2020).

### 4.2. Royal Jelly

The antimicrobial mechanism of action of royal jelly is related to proteins and peptides as well as to 10-hydroxy-2-decanoic acid (10-HAD). Major royal jelly proteins (MRJP 2, 5, and 7) and jelleines (I, II, and III) are characterized by antimicrobial activity [33]. Proteins may interact with the anionic phospholipids of the cell membrane and collapse it since they are positively charged (lysine, arginine, and histidine) [33,73]. Jelleines may interact with bacterial membranes by hydrophobic residues [73]. Moreover, MRJP 2 and 4 induce damage and dysfunction of the cell wall and membrane. What is more, royalisin has a strong antimicrobial effect because of three intramolecular disulfide bonds between cysteine residue [33]. Royalisin is a homolog of the defensin-1 [32,74]. Royalisin and 10-HAD inhibit bacterial growth [74]. 10-HAD disrupts the cell surface and the expression of glucosyltransferases gtfB and gtfC [74]. Additionally, apolipophorin III-like protein and glucose oxidase present in royal jelly exhibit antibacterial activity. Apolipophorin III-like protein carries lipids into an aqueous environment by protein-lipid complexes, while glucose oxidase oxidizes glucose to hydrogen peroxide [73]. Interaction of antimicrobial proteins (AMP) with cell membrane may determine permeabilization by three different models [73]:

→ Barrel-stave model: pores are formed in the hydrophobic core of the membrane by a circular assembly of AMPs (“hydrophobic domains of AMP pointing toward the lipid chains of the membrane, while the hydrophilic domains toward the interior of the pore”);
→ Carpet-like model: “The AMPs initially interact with the external surface of the membrane; subsequently, the charged region of the peptide interacts with the anionic phospholipids forming a carpet, which extends on the surface of the target membrane”. Thus, the model reduces lipid layer surface and membrane disruption.
→ Toroidal pore model: pores are formed in a membrane like in the barrel-stave model, but “the phospholipids assumed a completely curvature as a double layer”. Thus, “lines of the double layer become a continuous structure, with the consequent formation on a pore.”

Moreover, AMP may not only bind DNA, RNA, and proteins but also interfere with bacterial cytokinesis by cell filamentation [73].

### 4.3. Bee Venom

**Figure 2:**
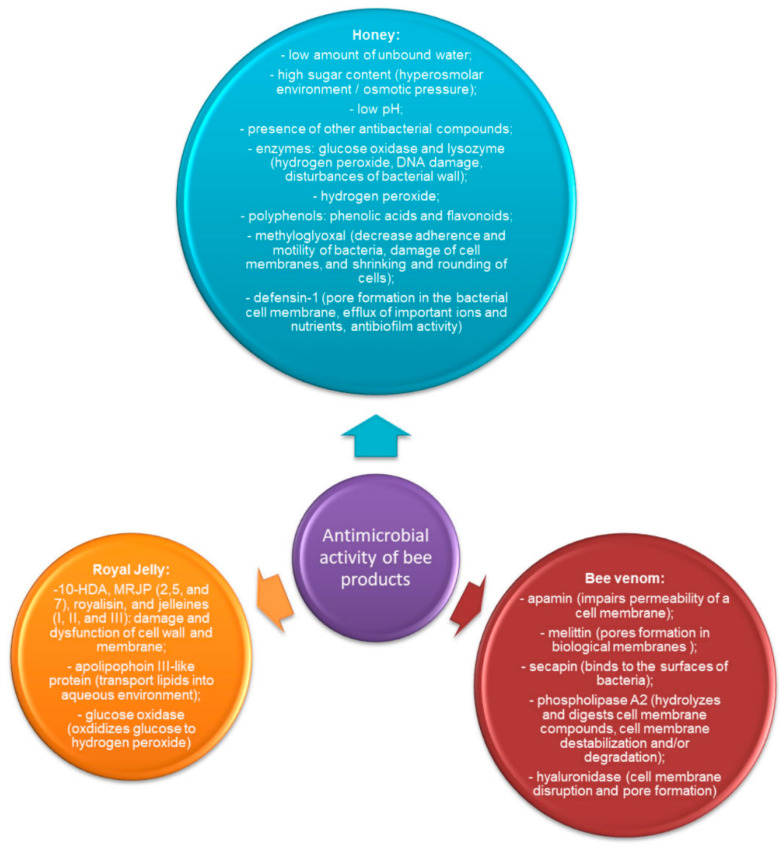
The summarized antimicrobial activity of honey, royal jelly, and bee venom.

In the case of bee venom, apamin, melittin, secapin, phospholipase A2, and hyaluronidase are responsible for its antimicrobial activity [74]. Melittin and phospholipase A2 are the main antimicrobial proteins in bee venom [75]. Melittin exhibits nonspecific cytolytic activity by interfering with biological membranes and forming pores in biological membranes [74,75]. Melittin has a hydrophobic section and positive charge, due to which it is attracted by the negatively charged membrane lipids and can embed itself in the membrane, which can lead to membrane fluctuations [75]. Melittin binds to membranes as monomers, but depending on its concentration, it may induce either transient or stable pores. In the case of transient pores, only ions may diffuse through the membrane, while in the case of stable pores the membrane becomes permeable to relatively large molecules (glucose) [76]. Finally, some phospholipids are pulled out by melittin, the asymmetry between two layers of the membrane occurs, and the membrane pressure changes, and the energy needed for the insertion of melittin is reduced. In consequence, transient pores in the membrane lead to cell lysis by the aggregation of melittin [75]. Apamin may also impair the permeability of the cell membrane for K^+^ ions by blocking calcium-activated K^+^ channels [76]. Secapin is a serine protease inhibitor-like peptide with anti-fibrinolytic, antielastolytic, and antibacterial activities, which may bind to the surfaces of bacteria [74,75]. Phospholipase A2 (PLA2) hydrolysis of the sn-2 fatty acyl ester bond of the membrane glycerol-3-phospholipids leads to the fatty acids and lysophospholipids liberation. Thus, the enzyme is able to hydrolyze and digest cell membrane compounds, destabilize cell membrane, and/or degrade it [75]. Antibacterial activity of phospholipase A2 towards gram-positive bacteria is related to the hydrolysis of bacterial membrane phospholipids and inhibition of the catalytic activity of PLA2. Since the phospholipid bilayer of gram-positive bacteria contains peptidoglycan, PLA2 is able to penetrate the cell membrane. The preferred site of PLA2 action is the bacterial envelope engaged in cell growth. Moreover, the cationic properties of the PLA2 molecule and the polyanionic properties of (lipo)teichoic acids in the bacterial cell wall promote the PLA2 attack of membrane phospholipids. In contrast, gram-negative bacteria are coated with the peptidoglycan layer and the outer membrane; thus, PLA2 is not able to penetrate the lipopolysaccharide envelope. The lipopolysaccharide-rich layer has to be disrupted first by other compounds, such as bactericidal/permeability-increasing proteins or the membrane attack complex of complement, before PLA2 hydrolyzes the phospholipid of the bacterial cell membrane [77]. Interestingly, membrane phospholipids may be exposed to the catalytic sites of PLA2 by opening melittin-induced channels [76]. Another enzyme present in bee venom is hyaluronidase, which is also responsible for cell membrane disruption and pore formation [75,78]. The summarized mechanism of action of honey, royal jelly, and bee venom is presented in Figure 2.

## 5. Comparison of Antimicrobial Properties of Bee Venom, Honey, and Royal Jelly

**Figure 3:**
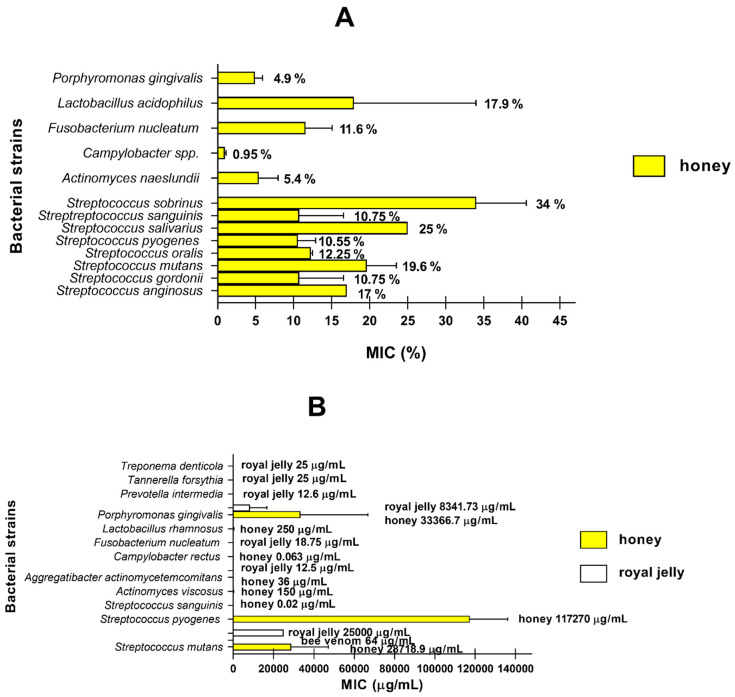
Antibacterial activity of honey, royal jelly, and bee venom. (**A**) The graph shows mean MIC values ± SEM for honey and royal jelly expressed in % for the bacterial strains causing oral cavity infections. Only experiments in which the MIC value was determined in % were included in the analysis [5,41,43,44,46,48,49,52,54,56,58,59,60]. (**B**) The graph shows mean MIC values ± SEM for honey, royal jelly, and bee venom expressed in µg/mL for the bacterial strains causing oral cavity infections. Only experiments in which the MIC value was determined in µg/mL were included in the analysis [45,47,51,53,57,63,64,65,67].

Taking into account the MIC values for different microorganisms obtained during the analysis of the literature, we prepared Figure 3A,B, which depicts mean values of MIC presented in % and µg/mL.

The analysis of average MIC values presented in [%] (Figure 3A) showed that honey had the lowest MIC values and exhibited the highest antimicrobial activity against *Campylobacter* spp., *P. gingivalis,* and *A. naeslundii*. Interestingly, *S. sobrinus* (average MIC: 34%) was the most resistant to different types of honey. In the case of MIC values expressed in µg/mL (Figure 3B), the highest antimicrobial activity was noticed for *C. rectus* and *S. sanguinis*. Strong activity of honey is also observed against *A. viscous*, *A. actinomycetemcomitans,* and *L. rhamnosus.* It is noteworthy that *P. gingivalis*, *S. pyogenes,* and *S. mutans* (average MIC: 33.4–117.3 mg/mL) were the most resistant to different types of honey. The analysis of royal jelly indicated that it exhibited the highest antibacterial activity against *A. actinomycetemcomitans*, *P. gingivalis*, *P. intermedia*, *F. nucleatum*, *T. forsythia,* and *T. denticola*, while the most resistant to royal jelly is *S. pyogenes*. Bee venom exhibited strong antibacterial activity only against *S. mutans*. Moreover, the presented results confirm that honey and royal jelly may be used in the treatment of oral cavity bacterial infections, such as periodontitis, gingivalis, caries, RAS, and pharyngitis. Interestingly, bee venom may be used in the treatment of caries and supragingival plaque.

The different MIC values observed for the same genus of bacteria may be caused by the difference in the composition and concentration of active substances in honey and royal jelly, the sensitivity of the analyzed strains, different durations of action of bee products on the tested microorganisms, and the method used to evaluate bioactivity [79]. Thus, obtaining the standardized antibacterial level of a bee product is necessary. To achieve this, a great many issues have to be taken into consideration [80]:

→ identification of the geographical origin: carbohydrates, proteins, and amino acids concentration and/or ratio depend on the origin of bee product;
→ the content of the nutritional components (different in each bee product): ingredients in the highest content (honey: carbohydrates 95–97% of its dry weight; royal jelly: 60–70% (*w*/*w*) water, dry matter—protein (27–41%), carbohydrates (~30%), lipids (8–19%); bee venom: peptides 48–50% of dry venom);
→ the storage condition (different for each bee product): honey (4 or 20 °C), royal jelly (4 °C for raw royal jelly, room temperature for lyophilized royal jelly, and 4–8 or <−18 °C for different days);
→ drying techniques (essential for storage), e.g., royal jelly (freezing);
→ different assessment methods of bee products, e.g., using the matrix-assisted laser desorption/ionization mass spectrometry (MALDI-MS) for detection of honey adulteration (based on oligosaccharide and polysaccharide profiles);
→ the techniques and standards of the bee product quality control (water content, microbial quality control, contaminant content);
→ the content of components exhibiting antibacterial activities (possible with different techniques: SDS-PAGE, MALDI-TOF-MS, LC-MS, HPLC, or Western blot).

All the above-mentioned issues are well described in the review by Luo et al. (2021) [80].

## 6. Conclusions

This review shows that natural products, such as honey, royal jelly, and bee venom, are very promising for the treatment of oral cavity bacterial infections. It is possible because the products exhibit strong antibacterial activity against the bacterial strains causing caries, periodontitis, gingivitis, pharyngitis, RAS, supragingival, and subgingival plaque. Moreover, these products are safer (since they do not have so many serious side effects) in comparison to the medicines approved for the treatment and/or prevention of oral cavity bacterial infections. The most important issue is to standardize the composition of bee products because their antimicrobial activity depends on their chemical composition and concentration of active compounds, which are determined by the origin of the product. Thus, more in-vitro and in-vivo studies should be performed to determine the exact composition and possible side effects of bee products, which will make them a safe and promising alternative to the drugs used today.

## Figures and Tables

**Table 1:** All diseases of the oral cavity, medications approved for the treatment/prevention, and possible side effects of treatment.

Disease	Bacteria	Medications Approved for the Treatment/Prevention	Side Effects
Caries	*Streptococcus mutans* [5,6], *Streptococcus salivarius* [3,5], *Streptococcus mitis* [6], *Streptococcus sobrinus* [5,6], *Streptococcus sanguinis* [3], *Lactobacillus* spp. [6], *Streptococcus anginosus* [5], *Streptococcus oralis* [5,6]	Fluoride [7]	Rare but serious: allergic reactions (hives, difficulty breathing, swelling of the face, lips, tongue, or throat), upset stomach, nausea and vomiting, staining, pitting, or any other changes in the appearance of teeth [7].
		Sodium fluoride and potassium nitrate [7]	Rare but serious: allergic reactions (rash, hives, itching, red, swollen, blistered, or peeling skin with or without fever, wheezing, tightness in the chest or throat, difficulty breathing, swallowing, or talking, unusual hoarseness, swelling of the mouth, face, lips, tongue, or throat), upset stomach or nausea [7].
Periodontitis	*Campylobacter* spp. [8,9], *Fusobacterium nucleatum* [9], *Porphyromonas gingivalis* [8,9,10], *Actinomyces actinomycetemcomitans* [8,9], *Tannerella forsythia* [9], *Porphyromonas intermedia* [8,9], *Treponema denticola* [8,9], *Streptococcus gordonii* [11]	Chlorhexidine [7]	Rare but serious: allergic reactions (hives, severe skin rash, wheezing, difficulty breathing, cold sweats, feeling light-headed, swelling of the face, lips, tongue, or throat) [7]. Common: mouth irritation, tooth staining, dry mouth, unusual or unpleasant taste in your mouth, or decreased taste sensation [7].
		Doxycycline [7,12], Minocycline [7]	Serious: allergic reactions (hives, difficulty breathing, swelling of the face or throat), severe skin reactions (fever, sore throat, burning in the eyes, skin pain, red or purple skin rash that spreads and causes blistering and peeling), fever, swollen glands, flu-like symptoms, muscle aches, severe weakness, unusual bruising, yellowing of the skin or eyes (increased sensitivity of the skin to sunlight—rare with minocycline) [7]. Common doxycycline side effects: nausea, vomiting, upset stomach, loss of appetite, mild diarrhea, skin rash, itching, darkened skin color, vaginal itching or discharge [7] Common minocycline side effects: numbness, tingling, burning pain, hair loss, skin or nails discoloration, dizziness, spinning sensation, muscle or joint pain, nausea, diarrhea, loss of appetite, swollen tongue, cough, trouble swallowing, rash, itching, or headache [7].
		Tetracycline [12]	More common: increased sensitivity of skin to sunlight, cramps or burning of the stomach, diarrhea [7]. Rare: abdominal pain, bulging fontanel (soft spot on head) of infants, headache, loss of appetite, nausea and vomiting, visual changes, yellowing skin [7]. Less common: itching of the rectal or genital (sex organ) areas, sore mouth or tongue [7].
		Carbamide peroxide [7]	Rare but serious: signs of an allergic reaction (rash, hives, itching, red, swollen, blistered, or peeling skin with or without fever, wheezing, tightness in the chest or throat, trouble breathing, swallowing, or talking, unusual hoarseness, or swelling of the mouth, face, lips, tongue, or throat), bad irritations for which carbamide peroxide liquid is used [7].
		Metronidazole [12]	More common: agitation, back pain, blindness, blurred vision, burning, numbness, tingling, or painful sensations in the hands or feet, changes in speech patterns, confusion, decreased vision, depression, dizziness, drowsiness, eye pain, fever, headache, irritability, lack of coordination, nausea, seeing or hearing things that are not there, seizures, shakiness and unsteady walk, slurred speech, stiff neck or back, trouble speaking, unsteadiness, trembling, or other problems with muscle control or coordination, unusual tiredness or weakness, vomiting, weakness in the arms, hands, legs, or feet [7]. Less common: black and tarry stools, blood in the urine, body aches or pain, burning while urinating, chills, clumsiness or unsteadiness, difficulty breathing, ear congestion, fainting, feeling of pelvic pressure, frequent or painful urination, irregular heartbeat, loss of voice, nasal congestion, pinpoint red spots on the skin, runny nose, skin rash, hives, redness, itching, sneezing, stomach and back pain (severe), unusual bleeding or bruising vaginal irritation, discharge, or dryness not present before taking the medicine [7].
		Ampicillin [12]	Common: eosinophilia, skin rash, diarrhea, nausea, swelling and pain, exanthema and enanthem in the oral region [7].
Gingivitis	*Actinomyces naeslundii* [13], *Porphyromonas intermedia* [13,14], *Actinomyces viscosus* [15]	Chlorhexidine [7,16]	Described in periodontitis.
		Hydrocortisone [7]	Serious: allergic reactions (hives, difficulty breathing, swelling of the face, lips, tongue, or throat), severe rectal pain or burning, bleeding from the rectum, new or worsening rectal irritation, fever or other signs of infection, severe headaches, ringing in the ears, pain behind the eyes, rectal itching, burning, or other irritation, dryness or skin changes around the rectum [7].
		Mouthrinse with antibacterial agents (listerine, cetylpyridinium chloride, peroxyl, delmopinol), toothpaste with a prophylactic agent (stannous fluoride), and antibiotics (minocycline and doxycycline) [16]	Dry mouth, bad taste, bad breath, tooth staining, calculus formation, unusual taste sensations, temporary taste alteration, redness, pain, tingling, numbness, gum irritation, periodontitis, tooth problems, pain, gum discomfort, toothache, periodontal abscess [16].
Pharyngitis	*Streptococcus pyogenes* [17]	Macrolides [7]	Serious: allergic reactions (hives, difficulty breathing, swelling in the face or throat) or severe skin reactions (fever, sore throat, burning eyes, skin pain, red or purple skin rash that spreads and causes blistering and peeling), fever, swollen glands, flu-like symptoms, muscle aches, severe weakness, unusual bruising, or yellowing of the skin or eyes [7].
		Glucocorticoids [7]	Serious: Allergic reactions (hives, difficulty breathing, swelling of the face, lips, tongue, or throat), blurred vision, eye pain, or seeing halos around lights, swelling, rapid weight gain, feeling short of breath, severe depression, feelings of extreme happiness or sadness, changes in personality or behavior, seizure (convulsions), bloody or tarry stools, coughing up blood, pancreatitis (severe pain in the upper stomach spreading to the back, nausea and vomiting, fast heart rate), low potassium (confusion, uneven heart rate, extreme thirst, increased urination, leg discomfort, muscle weakness or limp feeling), dangerously high blood pressure (severe headache, blurred vision, buzzing in the ears, anxiety, confusion, chest pain, shortness of breath, uneven heartbeats, seizure), sleep problems (insomnia), mood changes, increased appetite, gradual weight gain, acne, increased sweating, dry skin, thinning skin, bruising or discoloration, slow wound healing, headache, dizziness, spinning sensation, nausea, stomach pain, bloating, or changes in the shape or location of body fat (especially in the arms, legs, face, neck, breasts, and waist) [7].
		Cephalosporins [7,18]	Serious: allergic reaction to cephalexin (hives, difficult breathing, swelling in the face or throat), severe skin reaction (fever, sore throat, burning eyes, skin pain, red or purple skin rash with blistering and peeling), severe stomach pain, diarrhea that is watery or bloody (even if it occurs months after the last dose), unusual tiredness, feeling light-headed or short of breath, easy bruising, unusual bleeding, purple or red spots under the skin, a seizure, pale skin, cold hands, and feet, yellowed skin, dark colored urine, fever, weakness, or pain in the side or lower back, painful urination, diarrhea, indigestion, stomach pain [7], nausea, vomiting, and vaginal itching or discharge [7,18].
		Penicillins [7,18]	Serious: allergic reactions (hives, difficult breathing, swelling in the face or throat), severe skin reactions (fever, sore throat, burning eyes, skin pain, red or purple skin rash with blistering and peeling), pain, numbness, tingling, burning, or feeling cold, pale or mottled skin, blue-colored lips, fingers, or toes, severe pain, tingling, weakness, or swelling in the lower leg, weakness in the arms or legs, blistering, peeling, discoloration, or painful skin changes where the medicine was injected, severe stomach pain, diarrhea that is watery or bloody (even if it occurs months after the last dose), a light-headed feeling, as if you could pass out, slow heart rate, weak pulse, fainting, slow breathing (breathing may stop), pounding heartbeats or fluttering in the chest, confusion, agitation, hallucinations (seeing or hearing things that are not real), extreme fear, a seizure, pain, swelling, warmth, redness, bruising, bleeding, or a lump where the medicine was injected, easy bruising or bleeding, pale or yellowed skin, dark colored urine, urination problems, or signs of a new infection--fever, chills, mouth sores, warmth or redness under the skin, vaginal itching or discharge, diarrhea, itching, sweating, allergic reaction, flushing (sudden warmth, redness, or tingly feeling), feeling anxious, nervous, weak, or tired, headache, dizziness, drowsiness, muscle or joint pain, or pain, swelling, bruising, or a hard lump where an injection was given [7], nausea, vomiting [7,18].
Recurrent aphthous ulcers (RAS)	*Streptococcus sanguinis* [19]	Glucocorticoids and antimicrobial therapy (tetracycline and chlorhexidine gluconate) as topical pastes, mouthrinses, intralesional injections. Immunosuppressive, and anti-inflammatory (corticosteroids, dapsone, colchicine, thalidomide) [20]	Brown staining of the teeth and tongue, the possibility of increased numbers of oral yeast infections [20].
Supragingival plaque	*Streptococcus mutans* [21], *Streptococcus salivarius* [21], *Streptococcus mitis* [21], *Lactobacillus* spp. [21]	Not found	
Subgingival plaque	*Porphyromonas gingivalis* [21], *Campylobacter* spp. [21], *Fusobacterium nucleatum* [21], *Aggregatibacter* *actinomycetemcomitans* [21], *Tannerella forsythia* [21], *Campylobacter* spp. [21], *Fusobacterium nucleatum* [21], *Porphyromonas gingivalis* [21], *Porphyromonas intermedia* [21], *Treponema denticola* [21].	Not found	

**Table 2:** Antibacterial activity of different types of honey.

Bacteria	MIC	Honey Samples	Reference
*Streptococcus pyogenes*	12.5% (*w*/*v*)	Tualang honey	[43]
	11.25% (*w*/*v*)	Manuka honey	
	22.38% (*w*/*v*)	Coniferous honey	[44]
	21.78% (*w*/*v*)	Thyme honey	
	27.74% (*w*/*v*)	Citrus honey	
	1.25% (*w*/*v*)	Two types of stingless bee honey produced by *S. bipunctata* (SB) and *Scaptotrigona postica* (SP)	[52]
	8.00 ± 0.33 mg/mL	Acacia honey	[53]
	9.33 ± 0.22 mg/mL	Carob honey	
	9.83 ± 0.22 mg/mL	Eucalyptus honey	
	9.00 ± 0.00 mg/mL	Harmal honey	
	8.66 ± 0.22 mg/mL	Jujube honey	
	10.33 ± 0.22 mg/mL	Lavender honey	
	11.00 ± 0.33 mg/mL	Orange honey	
	10.50 ± 0.33 mg/mL	Reseda honey	
	8.50 ± 0.33 mg/mL	Rosemary honey	
	8.00 ± 0.00 mg/mL	Spurge honey	
	7.33 ± 0.22 mg/mL	Thyme honey	
	0.20 g/mL	13 different samples of Spanish honey	[63]
	0.25 g/mL	Multifloral honey H4a	
	0.10 g/mL	Multifloral honey H8a	
	13% (*w*/*v*) 15% (MIC_90_ *w*/*v*)	Tualang honey	[58]
	2% (*w*/*v*)	*F. nigra* honey, Tobago	[59]
	4% (*w*/*v*)	*F. nigra* honey, Trinidad	
	8% (*w*/*v*)	*M. favosa* honey, Tobago	
	16% (*w*/*v*)	*A. mellifera* honey, Tobago	
*Streptococcus mutans*	25%	Eucalyptus honey	[41]
	25%	Pincushion honey	[5]
		Fynbos honey	
		Bluegum honey	
		Manuka honey	
	100 μg/mL	Manuka 1 honey	[45]
	200 μg/mL	Manuka 2 honey	
	12.5–25%	Natural honey from a local grocery store in Jeddah (Saudi Arabia)	[46]
	32 μg/mL	Honey mouthwash	[47]
	1:5	Manuka honey 25+ NPA values	[51]
	2.5% (*w*/*v*)	Two types of stingless bee honey produced by *S. bipunctata* (SB) and *Scaptotrigona postica* (SP)	[52]
	6.3–25% (*w*/*v*)	Manuka honey	[54]
	>50%	Swiss midland honey	[56]
	>50%	German lowland honey	
	10%	Manuka honey	
	6.2 ± 3.4% (*w*/*v*)	Citrus honey	[60]
	4.5 ± 1.8% (*w*/*v*)	*Saturja* spp. honey	
	6.25% (*w*/*v*)	Oregano and sage honey	
*Porphyromonas gingivalis*	1:32 μg/mL	Honey mouthwash	[47]
	5% (MIC_50_ *w*/*v*)	German multifloral blossoms honey	[49]
	2% (MIC_50_ *w*/*v*)	Manuka honey	
	1:10	Manuka honey 25+ NPA values	[51]
	6.3–25% (*w*/*v*)	Manuka honey	[54]
*Streptococcus sanguinis*	25%	Eucalyptus honey	[41]
	25%	Pincushion honey	[5]
		Fynbos honey	
		Bluegum honey	
		Manuka honey	
	1:512 μg/mL	Honey mouthwash	[47]
	1.25%	Swiss midland honey	[56]
		German lowland honey	
		Manuka honey	
*Streptococcus sobrinus*	25%	Eucalyptus honey	[41]
	25%	Pincushion honey	[5]
		Fynbos honey	
		Bluegum honey	
		Manuka honey	
	50%	Swiss midland honey	[56]
	50%	German lowland honey	
	20%	Manuka honey	
*Streptococcus anginosus*	17%	Eucalyptus honey	[41]
	17%	Pincushion honey	[5]
		Fynbos honey	
		Bluegum honey	
		Manuka honey	
*Streptococcus gordonii*	25%	Eucalyptus honey	[41]
	25%	Pincushion honey	[5]
		Fynbos honey	
		Bluegum honey	
		Manuka honey	
	1.25%	Swiss midland honey	[56]
		German lowland honey	
		Manuka honey	
*Streptococcus oralis*	12%	Eucalyptus honey	[41]
	12.5%	Pincushion honey	[5]
		Fynbos honey	
		Bluegum honey	
		Manuka honey	
*Streptococcus salivarius*	25%	Eucalyptus honey	[41]
	25%	Pincushion honey	[5]
	50%	Fynbos honey	
	25%	Bluegum honey	
		Manuka honey	
*Actinomyces actinomycetemcomitans*	32 μg/mL	Honey mouthwash	[47]
	1:25	Manuka honey 25+ NPA values	[51]
*Fusobacterium nucleatum*	6.3–25% (*w*/*v*)	Manuka honey	[54]
	25% (*w*/*v*)	Citrus honey	[60]
	5.9 ± 0.9% (*w*/*v*)	*Saturja* spp. honey	
	6.25% (*w*/*v*)	Oregano and sage honey	
*Campylobacter spp.*	0.8–1.1% (*v*/*v*)	Manuka honey	[42]
	1:16 μg/mL	Honey mouthwash	[47]
*Actinomyces viscosus*	100 μg/mL	Manuka 1 honey	[45]
		Manuka 2 honey	
*Lactobacillus rhamnosus*	100 μg/mL	Manuka 1 honey	[45]
	200 μg/mL	Manuka 2 honey	
*Actinomyces naeslundii*	10%	Swiss midland honey	[56]
	1.25%	German lowland honey	
	5%	Manuka honey	
*Lactobacillus acidophilus*	50%	Swiss midland honey	
	2.5%	German lowland honey	
	1.25%	Manuka honey	

**Table 3:** Antibacterial activity of royal jelly.

Bacteria	Royal Jelly	Reference
*Actinomyces actinomycetemcomitans*	12.5 μg/mL	[63,64]
*Fusobacterium nucleatum*	12.5 μg/mL	[63]
	25 μg/mL	[64]
*Porphyromonas gingivalis*	0.2 μg/mL	[63]
	25 μg/mL	[64]
*Prevotella intermedia*	0.2 μg/mL	[63]
	25 μg/mL	[64]
*Tannerella forsythia*	25 μg/mL	
*Treponema denticola*	25 μg/mL	

**Table 4:** Antibacterial activity of bee venom, apitoxin, melittin, and phospholipase A2.

Bacteria	Bee Venom	Apitoxin in Nature	Apitoxin Commercial	Melittin	Phospholipase A2	Associated Melittin/Phospholipase A2	Reference
*Streptococcus mutans*	64 μg/mL						[65]
		20 μg/mL	20 μg/mL	40 μg/mL		80 μg/mL	[66]
*Streptococcus salivarius*		20 μg/mL	20 μg/mL	10 μg/mL		10 μg/mL	
*Streptococcus sobrinus*		40 μg/mL	20 μg/mL	10 μg/mL		10 μg/mL	
*Streptococcus mitis*		40 μg/mL	20 μg/mL	10 μg/mL		10 μg/mL	
*Streptococcus sanguinis*		30 μg/mL	20 μg/mL	10 μg/mL		10 μg/mL	
*Lactobacillus casei*		20 μg/mL	20 μg/mL	4 μg/mL	400 μg/mL	6 μg/mL	

## Data Availability

Data sharing not applicable. Publicly available publications were analyzed in this study. All of the used publications can be found using references.

## Abbreviations

10-HAD	10-hydroxy-2-decanoic acid
AMP	antimicrobial proteins
CFU	colony-forming unit
DNA	deoxyribonucleic acid
H_2_O_2_	hydrogen peroxide
HPLC	high-performance liquid chromatography
LC-MS	liquid chromatography–mass spectrometry
MALDI-TOF-MS	MALDI coupled to time-of-flight mass spectrometry
MBC	minimum bactericidal concentration
MGO	methylglyoxal
MIC	minimum inhibitory concentration
MLC	minimum lethal concentration
MRJP	main royal jelly protein
NPA	non-peroxide activity
PLA2	phospholipase A2
RAS	recurrent aphthous ulcers
RNA	ribonucleic acid
SDS-PAGE	Sodium Dodecyl Sulfate–PolyAcrylamide Gel Electrophoresis
SEM	standard error of mean
TH	Tualang honey
WHO	World Health Organization
*w*/*v*	weight/volume
